# Socioeconomic disparities in prehospital stroke care

**DOI:** 10.1186/s13049-019-0630-6

**Published:** 2019-05-02

**Authors:** Amanda Niklasson, Johan Herlitz, Katarina Jood

**Affiliations:** 1 Institute of Neuroscience and Physiology, Department of Clinical Neuroscience, The Sahlgrenska Academy, University of Gothenburg, Blå Stråket 7, plan 3, SE-413 45 Gothenburg, Sweden; 2 PreHospen – Centre for Prehospital Research, Faculty of Caring Science, Work Life and Social Welfare, University of Borås, Borås, Sweden

**Keywords:** Stroke, Transient ischaemic attack, Income, Education, Prehospital delay

## Abstract

**Background and purpose:**

Recent studies have revealed socioeconomic disparities in stroke outcomes. Here, we investigated whether prehospital stroke care differs with respect to socioeconomic status (SES).

**Methods:**

Consecutive stroke and TIA patients (*n* = 3006) admitted to stroke units at Sahlgrenska University Hospital, Gothenburg, Sweden, from 1 November 2014 to 31 July 2016, were included. Data on prehospital care were obtained from a local stroke register. Socioeconomic status was classified according to the average level of income and education within each patient’s neighbourhood (postcode area).

**Results:**

The median system delay from calling the emergency medical communication centre (EMCC) to start of brain computed tomography on hospital arrival was 3 h 47 min (95% confidence interval (CI) 3 h 30 min to 4 h 05 min) for patients within the lowest SES tertile and 3 h 17 min (95% CI 3 h 00 min to 3 h 37 min) for the highest tertile (*p* < 0.05). Patients with a lower SES were less likely to receive the highest priority in the ambulance (p < 0.05) and had lower rates of prehospital recognition of stroke/TIA (p < 0.05) than those with a high SES. No inequities were found concerning EMCC prioritisation or the probability of ambulance transport.

**Conclusions:**

We found socioeconomic inequities in prehospital stroke care which could affect the efficacy of acute stroke treatment. The ambulance nurses’ ability to recognise stroke/TIA may partly explain the observed inequities.

## Introduction

It is well known that socioeconomic status (SES) is a major determinant of health, quality of life and mortality. Socioeconomic inequities relating to disability and survival following stroke have been reported from several studies, both between and within countries, including high-income countries [1–8]. Inequities in received stroke care have been shown throughout the entire care chain [4, 6–12], including the probability of receiving reperfusion therapy [4, 10–12]. These inequities may contribute to the observed disparities in stroke outcome.

The time from stroke onset to hospital arrival is a crucial factor for timely acute stroke treatment and it is consequently a major determinant of stroke outcome. Associations between SES and prehospital delay have been reported [9, 13–17], with a longer delay for patients with a low SES, although some other studies [18–23] found no association. Moreover, studies have reported varying results relating to the probability of ambulance use [9, 19, 24, 25] in different socioeconomic groups, while prehospital priority levels and prehospital recognition of stroke and TIA by ambulance personnel have rarely been examined. The evidence relating to the association between SES and system delay is therefore inconclusive and knowledge of the underlying causes of a prolonged delay in more disadvantaged populations is lacking.

In this study, we aimed to investigate the association between neighbourhood SES and system delay in the early chain of care for stroke and TIA. In a second step, we studied factors contributing to system delay, including ambulance use, prioritisation by the emergency medical communication centre (EMCC) and in the ambulance and prehospital recognition of stroke and TIA by the ambulance personnel.

## Methods

### Study population

In this study, we included all patients who had received care for stroke (defined as ICD-10 codes I61, I63 or I64) or TIA (defined as ICD-10 code G45) at stroke units at Sahlgrenska University Hospital, Gothenburg, Sweden, between 1 November 2014 to 31 July 2016, and had a postcode of residence within the catchment area of the hospital at diagnosis. Sahlgrenska University Hospital consists of three different hospitals; Sahlgrenska Hospital, Mölndal Hospital and Östra Hospital, all with their own stroke units. The catchment area of Sahlgrenska University Hospital comprises five municipalities with a total of about 700,000 inhabitants. All patients suffering a suspected stroke or TIA in these municipalities receive hospital care at Sahlgrenska University Hospital, according to regional guidelines.

### Variables

Väststroke is a local stroke register reflecting care throughout the entire care chain for all stroke and TIA patients who have been hospitalised at stroke units at Sahlgrenska University Hospital. The register complements the Swedish Stroke Register (Riksstroke) with further details covering the chain of care among patients suffering a stroke/TIA that are not reported in Riksstroke. One example is various aspects of the prehospital chain of care. For this study, data on age, sex, diagnosis of stroke or TIA, postcode of residence, date of stroke or TIA onset, date and time of calling the EMCC, priority given by the EMCC, ambulance transport, priority in the ambulance, prehospital recognition of stroke or TIA by the ambulance personnel and date and time for the start of the first brain CT scan on hospital arrival were obtained from Väststroke.

### Classification of socioeconomic status

We used neighbourhood-level SES, defined by a combined measurement including average level of income and education for the residents in each postcode number within the primary catchment area of Sahlgrenska University Hospital. In addition to individual SES, evidence indicates that the average level of SES within a person’s neighbourhood is an additional and distinct predictor of health [26, 27]. Data on levels of income and education within each postcode area were obtained from Statistics Sweden on 7 June 2016. The most recently completed datasets were obtained, reflecting level of education collected in 2015, while information on income was collected for 2014. For education, data were collected among citizens aged 25–64 years, while data on income from employment were collected among those above 20 years of age. Level of education was defined as low, if the highest completed education was elementary school or upper secondary school, while a high level of education was defined as post-secondary education. Income was defined as low, if the income was less than the average income in Sweden, and high, if above average.

In all, the catchment area of Sahlgrenska University Hospital included 588 postcode areas with between three and 3294 inhabitants (mean 1188), where a minority of the postcode areas had fewer than 100 inhabitants (*n* = 31). In the first step, the average levels of income and education were examined separately for all postcode areas in the catchment area of the hospital. The postcode areas were ranked into deciles based on the proportions of citizens with low and high levels of income/education respectively, resulting in a total ranking from 2 to 20 for each of the two determinants of SES. A low ranking indicated a large proportion of citizens with low income/education and a small proportion with high income/education within the postcode area and vice versa. By combining the total ranking for the level of education with the total ranking for the level of income, a combined socioeconomic measurement ranging from 4 to 40 was obtained. In the next step, the combined socioeconomic ranking was used to divide the postcode numbers into tertiles and classify them as low SES (rank 4–17), intermediate SES (rank 18–28) or high SES (rank 29–40). In the last step, each patient was linked to the level of SES of his/her own postcode of residence. In 2015, 702,091 people lived within the catchment area of Sahlgrenska University Hospital, of which 241,324 (34.4%) lived in a postcode area classified as having a low SES, 252,857 (36.0%) lived in neighborhoods with an intermediate SES and 207,910 (29.6%) people lived in postcode areas with a high SES.

### Statistical methods

System delay, defined as the time interval between the patient’s or a bystander’s alarm call to the EMCC and start of the first performed brain CT scan on hospital arrival, was calculated. Data on priority levels made by the EMCC and in the ambulance were dichotomised so that those who received the highest priority (priority 1) formed one category and those with a lower priority the other. In Sweden, the EMCC prioritize patients into one of three priority levels based on the Swedish index for emergency medical alarm reception. Priority 1 is given to potentially acute life-threatening conditions, priority 2 to acute but not life-threatening conditions and priority 3 to conditions needing medical evaluation but where equitable waiting time should not affect the outcome. The dichotomization was done because Swedish guidelines state that patients with a suspected stroke or TIA should be given priority 1 by the EMCC and in the ambulance.

**Fig. 1:**
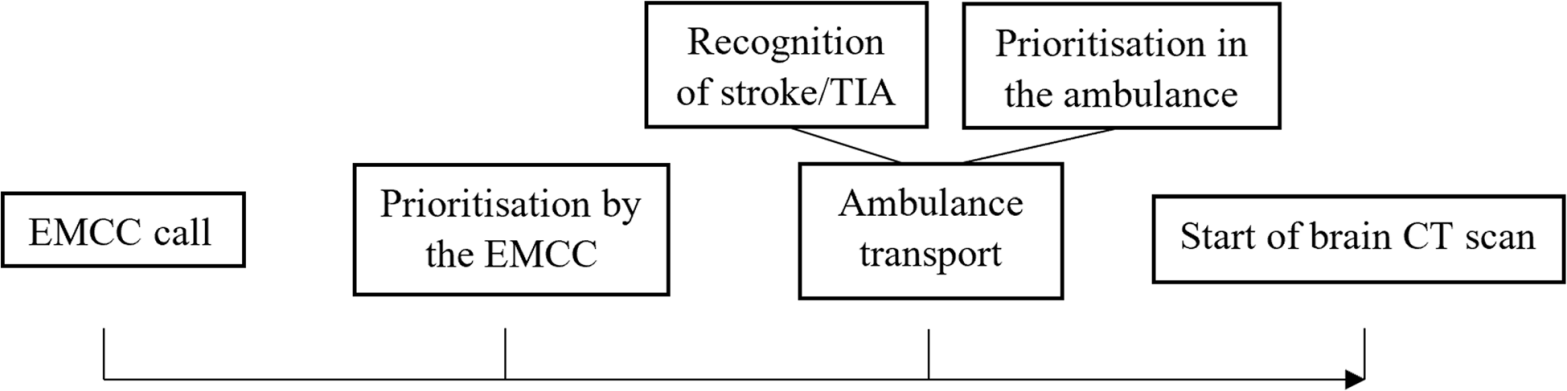
Flowchart describing the pathway from EMCC call to start of brain CT scan on hospital arrival. EMCC indicates emergency medical communication centre

The impact of SES on system delay was analysed among patients who had been transported by ambulance, using linear regression with a log-transformed response. The estimated marginal means predicted by the model were used to investigate the median system delay in hours and minutes for each socioeconomic group. The time variable was log-transformed using the natural logarithm, before the linear regression was performed because of its non-normal distribution, resulting in the median system delay after calculating the antilogarithms of the estimated marginal means. Binary logistic regression was used to investigate the associations between SES and prioritisation by the EMCC, ambulance use, prioritisation in the ambulance and probability of prehospital recognition of stroke/TIA. The results of these analyses are presented as odds ratios (OR) and 95% confidence intervals (CI). Both univariable and multivariable analyses were performed for each outcome. The multivariable analyses for system delay, prioritisation by the EMCC, ambulance use and prioritisation in the ambulance were adjusted for age, sex, and stroke/TIA, while the analysis of prehospital recognition of stroke and TIA was adjusted for sex and age. Figure 1 shows a summary of the analysed components of delay from EMCC call to performed CT scan. In all the analyses, a low SES was used as a reference. In addition, we investigated whether there was an association between SES and the different outcomes using the continuous ordinal SES scale (score 4–40). All the statistical analyses were conducted using IBM SPSS Statistics 24. Statistical significance was assumed at a *p*-value of < 0.05.

## Results

**Fig. 2:**
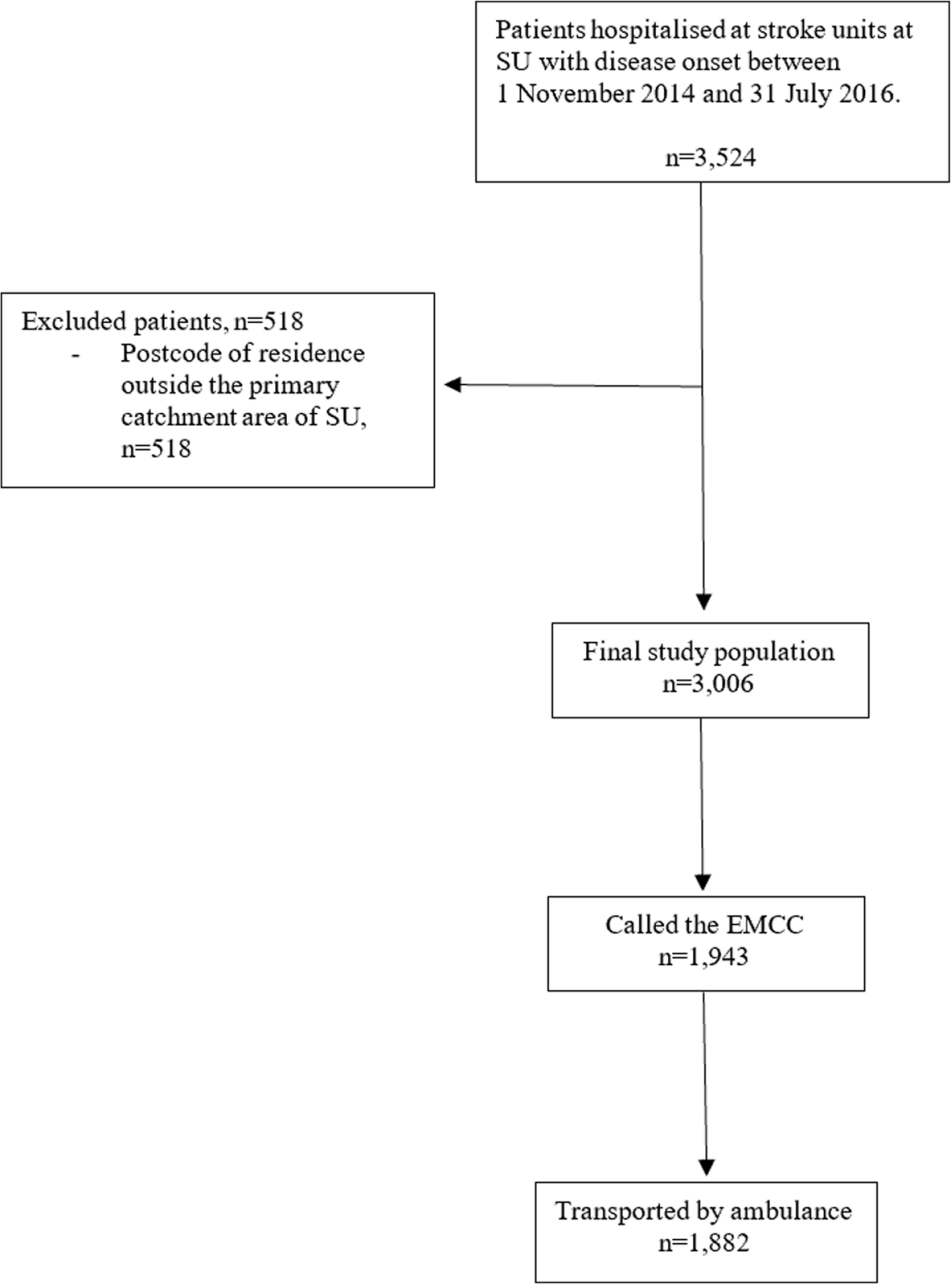
Flowchart describing the selection of included patients. SU indicates Sahlgrenska University Hospital. EMCC indicates emergency medical communication centre

During the study period, a total of 3524 patients were admitted to stroke units at Sahlgrenska University Hospital with an event of stroke or TIA between 1 November 2014 and 31 July 2016. After the exclusion of patients whose postcodes of residence were outside the catchment area of the hospital, the final study population consisted of 3006 patients. Figure 2 shows a flowchart of the included patients. Of these, 1943 (64.6%) had contacted the EMCC and were included in the analysis of prioritisation by the EMCC, while 1882 (62.6%) had been transported by ambulance to hospital and were included in the analyses of system delay, prioritisation in the ambulance and probability of prehospital recognition of stroke or TIA. Table 1 shows the baseline characteristics of the study population. Stroke was the discharge diagnosis in 2219 (73.8%) cases, while 787 (26.2%) had a TIA as the cause of hospitalisation. In all, 1155 (38.4%) were classified as having a low SES, 1088 (36.2%) an intermediate SES and 763 (25.4%) a high SES.

**Table 1:** Baseline characteristics of the study population

Variable	Total study population (*n* = 3006)
Age (mean ± SD)	75.4 ± 13.1
Female sex, n (%)	1479 (49.2)
Low SES, n (%)	1155 (38.4)
Intermediate SES, n (%)	1088 (36.2)
High SES, n (%)	763 (25.4)
Stroke, n (%)	2219 (73.8)
Contacted EMCC, n (%)	1943 (64.6)
Transported by ambulance, n (%)	1882 (62.6)

SES indicates socioeconomic status and EMCC indicates emergency medical communication centre

No statistically significant socioeconomic disparities were found in terms of prioritisation at the EMCC, in either univariable or multivariable analyses, regardless of using the three ordinal categories or the continuous ordinal scale. Of the 1943 patients who had contacted the EMCC, 1855 (95.5%) had registered data on priority received. Of these, 1311 (70.7%) received the highest priority level. The multivariable adjusted OR for priority 1 at the EMCC was 1.18 (95% CI 0.94–1.49) (*p* = 0.151) for patients with an intermediate SES, 1.23 (95% CI 0.95–1.59) (*p* = 0.122) for those with a high SES and a *p*-value of 0.098 for the continuous SES-scale. Likewise, the probability of arrival by ambulance on hospital was not associated with socioeconomic status, in either univariable or multivariable analyses. The multivariable adjusted OR for ambulance transport was 0.96 (95% CI 0.80–1.15) (*p* = 0.639) among patients living in postcode areas with an intermediate SES, 0.92 (95% CI 0.76–1.12) (*p* = 0.399) for those with a high SES and *p* = 0.270 for the continuous SES scale.

**Fig. 3:**
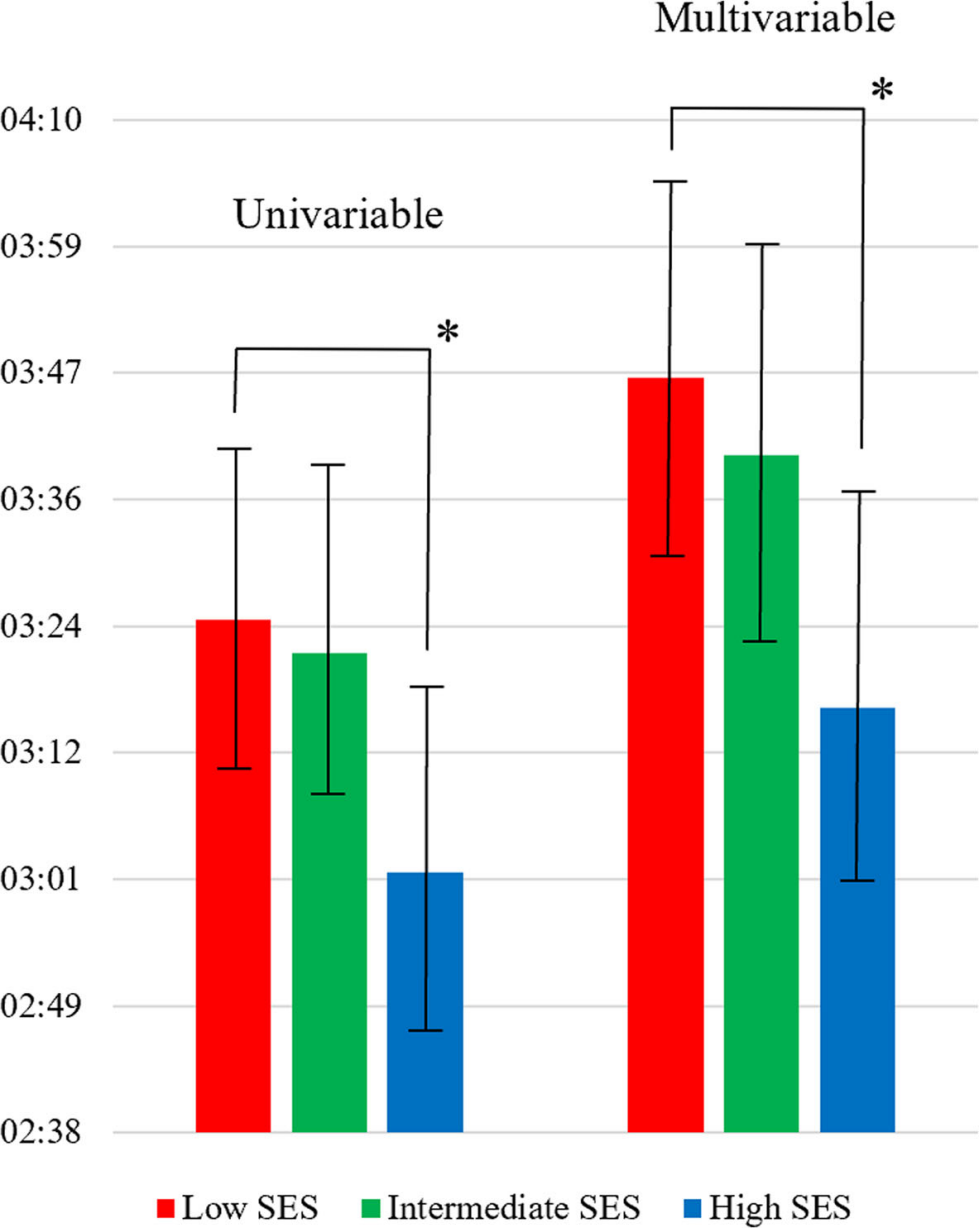
Univariable and multivariable adjusted median system delay (hours:minutes from calling EMCC to start of brain CT scan on hospital arrival) with 95% confidence intervals depending on socioeconomic status. The multivariable analysis was adjusted for sex, age and stroke or TIA as the discharge diagnosis. SES indicates socioeconomic status. * *p*-value < 0.05

In all, 1786 (94.9%) of the 1882 patients who had been transported by ambulance had registered data on both the time of EMCC call and the time of the start of brain CT scan on hospital arrival. Socioeconomic inequities were observed with respect to system delay in both univariable and multivariable analyses, see Fig. 3. In the univariable analysis, patients with a low SES had a median system delay of 3 h 25 min (95% CI 3 h 11 min-3 h 40 min), those with an intermediate SES 3 h 22 min (95% CI 3 h 08 min-3 h 38 min) (*p* = 0.811) and those with a high SES 3 h 02 min (95% CI 2 h 47 min-3 h 19 min) (*p* = 0.043). There was also a significant association with the continuous SES scale (*p* = 0.034). After adjustments for sex, age and stroke or TIA as the discharge diagnosis, patients with a low SES had a median system delay of 3 h 47 min (95% CI 3 h 30 min-4 h 05 min), those with an intermediate SES 3 h 40 min (95% CI 3 h 23 min-3 h 59 min) (*p* = 0.570), patients living in postcode areas with a high SES 3 h 17 min (95% CI 3 h 00 min-3 h 37 min) (*p* = 0.015), while the *p*-value for the continuous scale was 0.009.

Of the 1882 patients who had been transported by ambulance to hospital, 1800 (95.6%) had registered data on priority in the ambulance. Of these, 577 (32.1%) had been regarded as priority 1, while 1223 (67.9%) had received a lower priority. Priority in the ambulance showed a significant association with the continuous SES scale, in both univariable and multivariable analysis (*p* = 0.032 and *p* = 0.005 respectively). As shown in Table 2, in the univariable analysis, a statistically significant difference in the probability of receiving the highest priority was observed between the lowest and the highest SES groups. After adjustment, a socioeconomic gradient was found, where the probability of being given priority 1 increased as the level of SES became higher.

**Table 2:** Odds ratios and 95% confidence interval for receiving priority 1 in the ambulance

Socioeconomic status	Odds ratio (95% CI)	p-value	Adjusted odds ratio (95% CI)	*p*-value
Low (ref.)	1.00		1.00	
Intermediate	1.20 (0.95–1.50)	0.129	1.27 (1.01–1.62)	0.043
High	1.31 (1.02–1.69)	0.036	1.43 (1.10–1.87)	0.008

Multivariable analysis adjusted for sex, age and stroke/TIA

In all, 1789 (95.1%) of the 1882 patients who had been transported by ambulance had registered data on whether a stroke or TIA had been recognised by the ambulance personnel. A stroke or TIA had been recognised in 1363 (76.2%) cases, while 426 (23.8%) remained unrecognised until hospital arrival. In both univariable and multivariable analyses of the three ordinal categories of SES, the ambulance personnel recognised stroke and TIA to a significantly greater extent in patients with a high SES compared with those with a low SES, see Table 3. However, when using the continuous SES scale, the association did not reach the level of statistical significance (*p* = 0.065 and *p* = 0.066 for univariable and multivariable analyses respectively).

**Table 3:** Odds ratio and 95% confidence intervals for prehospital recognition of stroke or TIA by the ambulance personnel

Socioeconomic status	Odds ratio (95% CI)	p-value	Adjusted odds ratio (95% CI)	p-value
Low (ref.)	1.00		1.00	
Intermediate	1.07 (0.84–1.37)	0.589	1.08 (0.84–1.38)	0.567
High	1.45 (1.08–1.94)	0.013	1.44 (1.08–1.93)	0.014

Multivariable analysis adjusted for age and sex

## Discussion

In this study, we found socioeconomic inequities in prehospital stroke care. Patients living in postcode areas with a lower SES had a prolonged median system delay, a lower probability of receiving the highest priority in the ambulance and their diagnoses were less frequently recognised by the ambulance personnel as compared to those with a higher SES. No socioeconomic disparities were found in terms of the probability of ambulance transport or the prioritisation by the EMCC.

Patients with a low SES had a 30-min longer median system delay than those with a high SES. This delay could potentially affect the efficacy of acute stroke treatments and subsequently contribute to socioeconomic disparities in stroke outcomes [28, 29]. Three other studies [15, 18, 19] have designs similar to ours, investigating median time spans in the acute stroke care chain depending on SES. Two of them [18, 19], both comprising patients during the late 1990s, found no or only small clinically relevant socioeconomic disparities in prehospital delay. On the other hand, a Canadian study published in 2012 [15] found that patients with a low income had more than a one-hour longer median delay from stroke onset to hospital arrival than those with a high income. Other studies have used cut-off times to define delay. The most commonly used are hospital arrival beyond three or six hours from stroke onset, in which some studies have observed socioeconomic inequities [9, 14, 16, 17], while others have not [20, 22, 23]. One study [13] found socioeconomic disparities in prehospital delay when using two hours as the cut-off, while another study [21] reported no such disparities.

The inconsistent observations in these studies might be a result of differences in the definitions of SES, delay, study design and differences in social structures and welfare systems between the countries in which the research has been conducted. Many studies have approximated SES by using single SES indicators such as income or education alone. However, evidence from the literature indicates that SES is a complex measurement comprising a combination of social factors [27]. For example, education and income are not interchangeable, hopefully making a combination of the two a stronger indicator of SES than using only one of the variables [27]. Furthermore, neighbourhood SES might influence health and received care in ways other than the individual socioeconomic position [27]. In addition, divergent findings between older and more recent studies could be partly explained by changes in the guidelines for acute stroke care, as Alteplase was licensed for the treatment of acute stroke in the United States in 1996, in Canada in 1999 and in Europe in 2002 [29]. In line with Link and Phelan’s fundamental cause theory, there might be social inequalities in access to the newest and most modern treatments, where patients with a higher SES are more able to take advantage of the innovations, thereby resulting in increasing disparities over time [30].

Our findings also provide indications of possible contributory factors to socioeconomic inequities in system delay. Prehospital recognition of stroke and TIA, high prehospital priority levels and activating stroke alerts are fundamental to reducing delay and improving reperfusion rates [9, 20]. To the best of our knowledge, there are no previous studies analysing the association between SES and prehospital priority levels. In our study, a socioeconomic gradient was observed, showing that the probability of receiving priority 1 in the ambulance decreased as the level of SES became lower. On the other hand, the level of SES was not associated with prioritisation by the EMCC. One possible explanation is that the EMCC generally gave the patients a higher priority than the ambulance personnel. Our findings of a lower probability of prehospital recognition of stroke and TIA among patients with a lower SES may potentially contribute to lower prioritisation both in the ambulance and in hospital and consequently prolonged system delay.

The socioeconomic inequities with respect to priority in the ambulance and recognition of stroke and TIA could be a result of discrimination. However, it is likely that factors other than those included in this study also affect received prehospital stroke care. Differences in the delay from symptom onset to calling the EMCC between socioeconomic groups could be one explanation. If the time from stroke onset exceeds the window of time for reperfusion therapy, this could contribute to lower prioritisation in the ambulance. Another explanation could be differences between the SES groups in their way of communicating their symptoms, which might facilitate or complicate prehospital recognition of stroke and TIA by the ambulance personnel. Differences in ethnicities and proportions of non-Swedish speaking residents between the SES-groups might be another contributory factor, as it has been shown to influence received stroke care in some studies [9].

Regardless of its causes, awareness of the existence of socioeconomic inequities in prehospital stroke care is one of the first steps in the work towards reducing these disparities. Another important intervention would be to improve stroke knowledge and awareness of the potential of conscious and unconscious bias. An improved ability to recognise stroke symptoms and atypical stroke presentations among medical personnel would be beneficial to all social groups and lower socioeconomic groups would probably benefit the most. Spreading knowledge of stroke/TIA in the general population might also be an important step to reduce prehospital delay. It has been shown that stroke knowledge is lower in more disadvantaged social groups [31–33]. In this study, 64.6% of stroke and TIA patients contacted the EMCC and 62.6% were transported by ambulance to the hospital, a result that might reflect a general low knowledge of stroke and TIA in the society. Nevertheless, the result is fully in line with the annual report for 2016 from the Swedish Stroke Register (Riksstroke), reporting that 72% of stroke patients and 54% of TIA patients were transported by ambulance to hospital. Previous studies, conducted both in Sweden [9, 34] and abroad [18, 19], has shown a wide range in the use of emergency services among stroke and TIA patients, ranging from 35 to 76%.

The study has some strengths and some limitations. Data were retrieved from the local quality register Väststroke and comprised all the patients with a stroke or TIA diagnosis that had been hospitalised at stroke units at Sahlgrenska University Hospital and lived in the primary catchment area of the hospital. During the study period, almost all (> 95%) hospitalised stroke patients at Sahlgrenska University Hospital were admitted to a stroke unit according to the Swedish Stroke Register (Riksstroke). As a result, no exclusion was made based on social characteristics, resulting in a large unselected study population, reflecting the true socioeconomic diversity among hospital-treated stroke patients. Our study was a single-centre study, which eliminated the influence of socioeconomic disparities on access to highly specialised hospitals and differences in prehospital stroke care between university and non-university hospitals. On the other hand, the single-centre study design could influence the heterogenicity in the study population and thus limits the generalisability of the results. We chose to study the time from EMCC call to start of brain CT scan on hospital arrival, as it reflects the complete care chain from the patient’s or a bystander’s decision to seek medical care until potential acute treatments can be initiated. To the best of our knowledge, socioeconomic disparities in this time span have not previously been studied. Delays from hospital arrival to CT scan have previously been observed [4], which contributes to delays until the administration of acute treatments and it is important to include this in studies of this kind. Another strength of this study is the inclusion of both stroke and TIA patients. It is likely that some patients who received TIA as a discharge diagnosis had ongoing stroke symptoms during ambulance transportation and were candidates for admission as stroke alerts and then fully recovered after thrombolysis. However, we do not have data on the proportion of hospitalised TIA patients admitted to stroke units during the study period. According to regional guidelines, all TIA patients should be admitted to stroke units. As access to stroke unit care has been reported to be lower for groups with a low SES [35, 36], a potential selection bias would, if anything, result in an underestimation of the association between our measurements of prehospital care and SES. One limitation of this study was that only the date and not the time of stroke onset is recorded in the register. We were therefore unable to adjust for patients’ or bystanders’ delay in contacting the EMCC. However, reliable information on the exact time of stroke onset is difficult to obtain in unselected stroke populations. Neither did we have information on the distance between the ambulance stations and the patients’ locations at the time of disease onset, as the actual driving time might influence system delay. However, there are several ambulance stations and three different hospitals included in the catchment area of the Sahlgrenska University Hospital which should reduce such disparities. In addition, the differences in prioritisation in the ambulance and prehospital recognition of stroke/TIA are not explained by differences in driving distance. Another limitation might be that SES was approximated by linking the patients to the average level of SES within their postcode areas, as not all citizens within a neighbourhood have the same level of income and education. However, one great strength in our approximation of SES is the inclusion of both income and education in a combined measurement, better reflecting the complexity of SES than the use of single variables [27].

## Conclusions

This study shows that socioeconomic inequities exist in prehospital stroke care in Sweden. Patients with a lower SES have a 30-min longer delay from EMCC call to the start of brain CT scan on hospital arrival compared with patients with a high SES, a clinically significant delay that may potentially affect the efficacy of acute stroke treatment. The ambulance personnel’s ability to recognise stroke and TIA might be an important underlying factor for the observed socioeconomic disparities in prehospital stroke care, resulting in lower prioritisation in the ambulance and consequently prolonged system delay. Taken as a whole, these findings might contribute to previously observed socioeconomic disparities in stroke outcomes and are important targets for improvements aimed at realising the goal of delivering the best stroke care for all patients on equal grounds.

## Abbreviations

CI: Confidence interval
CT: Computed tomography
EMCC: Emergency medical communication centre
OR: Odds ratio
SES: Socioeconomic status
TIA: Transitory ischaemic attack
